# Early high‐dose aflibercept 8 mg clinical outcomes in wet age‐related macular degeneration patients resistant to aflibercept 2 mg or faricimab

**DOI:** 10.1111/aos.70213

**Published:** 2026-08-03

**Authors:** Pinja Sutinen, Idan Hecht, Raimo Tuuminen

**Affiliations:** ^1^ Helsinki Retina Research Group University of Helsinki Helsinki Finland; ^2^ Department of Ophthalmology Mayo Clinic Rochester Minnesota USA; ^3^ Department of Ophthalmology Mehiläinen Hospital Helsinki Finland; ^4^ Faculty of Health Sciences Ben‐Gurion University of the Negev Beer‐Sheva Israel; ^5^ Department of Ophthalmology Kymenlaakso Central Hospital Kotka Finland

Editor,

Intravitreal anti‐vascular endothelial growth factor (anti‐VEGF) injections to the retina have become the standard of care for wet age‐related macular degeneration (wAMD). Despite the overall success of intravitreal injections with agents such as ranibizumab or aflibercept 2 mg, many patients require frequent, long‐term treatment with limited possibility for interval extension. Aflibercept 8 mg, a high‐dose formulation of the previously widely used 2 mg aflibercept, was developed to provide more sustained VEGF suppression and enable longer injection intervals, potentially leading to improved visual and anatomical outcomes (Lanzetta et al., 2024). Although clinical trials have shown the efficacy and safety of aflibercept 8 mg (Korobelnik et al., 2026), real‐world evidence is needed to evaluate its outcomes and safety in routine practice, especially in previously treated wAMD patients with suboptimal response.

Here, in a prospective, single‐centre, real‐world observational cohort study we evaluated early visual, anatomical and safety outcomes of intravitreal aflibercept 8 mg (Eylea HD® 114.3 mg/mL solution, Bayer AG, Leverkusen, Germany) under a treat‐and‐extend regimen protocol in 38 consecutive patients with 38 pre‐treated wAMD eyes non‐responding to aflibercept 2 mg (Eylea® 40 mg/mL solution, Bayer AG) or faricimab (Vabysmo® 120 mg/mL solution, F. Hoffmann‐La Roche AG, Basel, Switzerland). Non‐response to prior anti‐VEGF adhered to criteria of our previous Current Care Guideline and RCT (Taipale et al., 2020; Tuuminen et al., 2017) as illustrated in Figure S1. Patients were enrolled in the study from March 2025 to September 2025 and followed up until June 2026 at the Department of Ophthalmology, Mehiläinen Hospital, Helsinki, Finland (Tables S1 and S2).

Mean central retinal thickness (CRT) decreased from 361.1 ± 91.7 μm at baseline to 243.8 ± 86.7 μm after induction with aflibercept 8 mg, 222.6 ± 60.7 μm at 6 months after the switch, and 205.5 ± 51.1 μm at final follow‐up (all *p* < 0.001 vs. baseline, Figure S2A). Best‐corrected visual acuity (BCVA) remained stable from 64.1 ± 14.0 ETDRS letters at baseline to 63.9 ± 14.2 letters at final follow‐up (*p* = 0.888, Figure S2B). Intraocular pressure remained stable from 11.5 ± 3.3 mmHg at baseline to 11.4 ± 3.2 mmHg at final follow‐up (*p* = 0.952, Figure S2C). Intraretinal fluid decreased from 53% at baseline to 13% after induction and 16% at final follow‐up (both *p* < 0.001, Figure 1a, Figures S2D and S3). Subretinal fluid decreased from 71% at baseline to 29% after induction and 32% at final follow‐up (both *p* < 0.001, Figure 1b, Figures S2E and S3). Mean treatment interval increased from 4.9 ± 1.3 weeks at baseline to 7.0 ± 1.4 weeks at final follow‐up (*p* < 0.001, Figure S2F). Linear regression analysis revealed the greater the baseline CRT, the more resolution of CRT was observed at final follow‐up timepoint (*R* ^2^ = 0.737, *p* < 0.001, Figure 1c). In a subgroup analysis, anatomical response to aflibercept 8 mg appeared similar regardless of the previously used anti‐VEGF therapy (Figure S4). Two adverse events occurred during follow‐up. One mild stroke occurred in a female patient aged 80 years previously treated with faricimab, 5 months after induction with aflibercept 8 mg. The patient fully recovered, and intravitreal injections were postponed for 2 months; drug and dose remained the same. In addition, one eye showed a significant decrease in BCVA, from 79 ETDRS letters at the time of switch to aflibercept 8 mg vs. 53 letters at 6 months and 45 letters at final visit, which was associated with progression of macular atrophy (Figure 1d).

**FIGURE 1:**
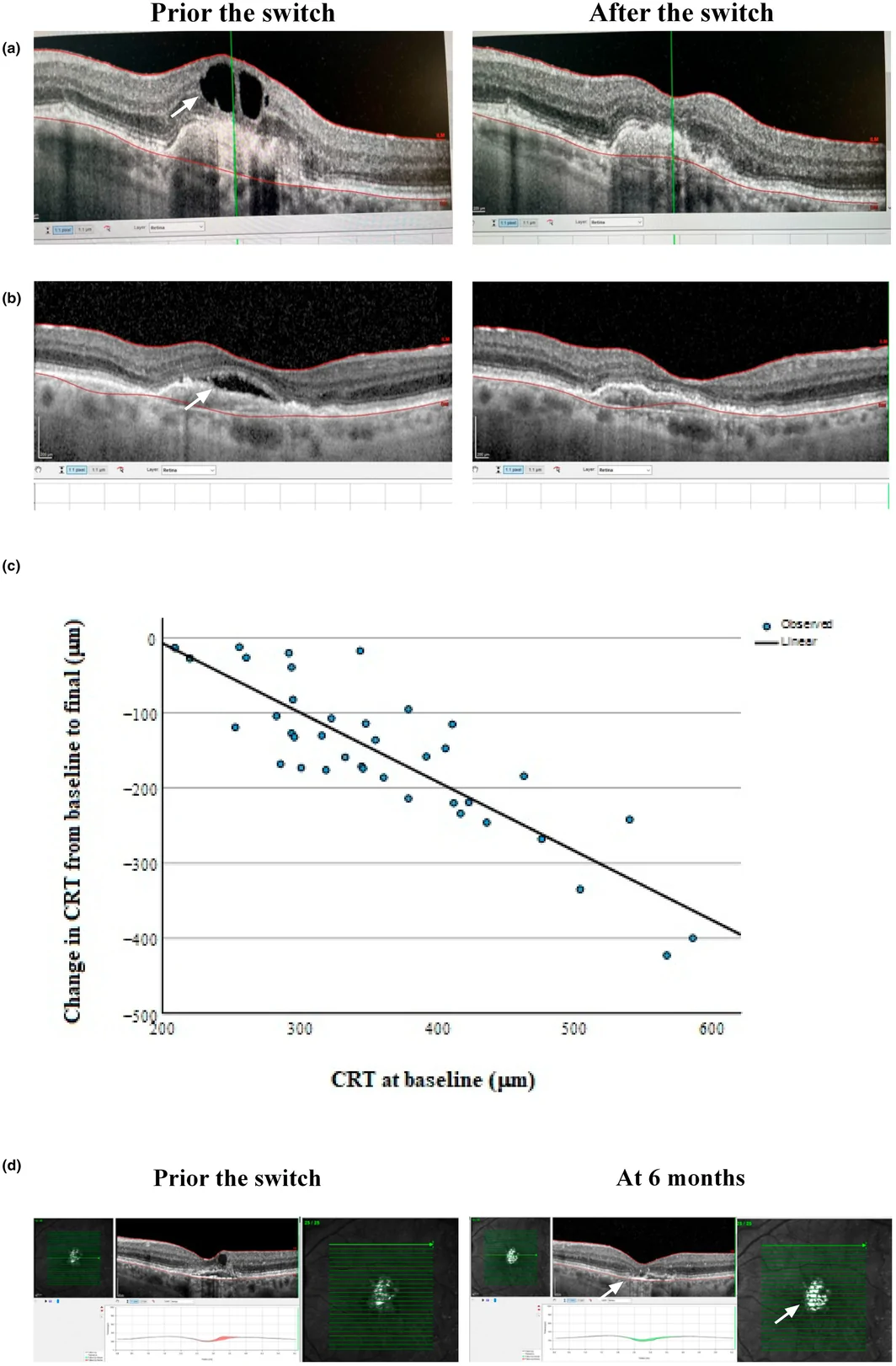
Early visual, anatomical and safety outcomes of intravitreal aflibercept 8 mg (Eylea HD® 114.3 mg/mL solution) in wet age‐related macular degeneration eyes non‐responding to aflibercept 2 mg (Eylea® 40 mg/mL solution) or faricimab (Vabysmo® 120 mg/mL solution. (a‐b) Representative optical coherence tomography scans prior and after the switch to aflibercept 8 mg. Arrows indicate resolution of (a) intraretinal fluid and (b) subretinal fluid. (c) Linear regression analysis between baseline CRT and change in CRT from baseline to final* follow‐up timepoint. (d) Optical coherence tomography scans of an eye with more than 10 ETDRS letter decline in BCVA. Representative scans (left side) prior the switch to aflibercept 8 mg and (right side) at 6 months. Arrows indicate progression of macular atrophy. *17 eyes had 9‐month, and 21 eyes had 1‐year follow‐up. BCVA = best‐corrected visual acuity, CRT = central retinal thickness.

Similar reductions in retinal fluid and CRT with extended dosing intervals have been reported in phase 2 and 3 trials evaluating aflibercept 8 mg in diabetic macular oedema and wAMD (Lanzetta et al., 2024). However, previous trials primarily included treatment‐naïve populations, in contrast to the pre‐treated non‐responder cohort in the present study. The combination of stable visual acuity and reduction in fluid presence suggests effective disease control as functional recovery in chronic wAMD is often not probable with pre‐existing fibrovascular and atrophic characteristics of the macula. A methodological distinction compared with many randomized clinical trials is that complete macular dryness was not required in this study, which aligned with our previous RCT on Eylea 2 mg (Taipale et al., 2020). Similar approaches are commonly used in treat‐and‐extend protocols in a real‐world setting (Freund et al. 2015). Prior studies have also demonstrated that individualized treatment strategies can maintain visual outcomes even in the presence of persistent or recurrent fluid, suggesting that complete dryness is not always required. Allowing longer dosing intervals is a worthwhile goal in reducing treatment burden and lowering healthcare resource utilization.

Several limitations should be considered. This was a prospective, single‐centre observational study with a relatively small sample size and heterogeneous prior treatment exposure. Especially subgroup analysis is likely underpowered, and residual confounders may exist. The absence of a control group limits causal inference. Follow‐up period was limited to early outcomes, and longer‐term durability of response and concerns regarding macular atrophy acceleration remain to be determined. Lack of standardized definition for non‐response to prior anti‐VEGF therapy may limit reproducibility and generalizability of the findings. In addition, complete macular dryness was not required before extension. Although this approach reflects real‐world management in many of the units, it may have introduced heterogeneity in treatment decisions and variability in anatomical outcome assessment. The present findings should be interpreted as preliminary and require confirmation in larger post‐marketing (phase IV) multicentre controlled studies.

## FUNDING INFORMATION

The study was supported by grants from the Friends of the Blind, the Finnish Cultural Foundation, the Finnish Eye Foundation, the Finnish Ophthalmological Society, the Maud Kuistila Memorial Foundation, the Nissi Foundation, the Paulo Foundation, the Waldemar von Frenckell Foundation and the HUS Specific Catchment Area (ERVA) Clinical Research Grants.

## CONFLICT OF INTEREST STATEMENT

Dr. Tuuminen is a scientific adviser (advisory board, honoraria) to Alcon Laboratories, Inc., Allergan, Inc., Bayer AG, F. Hoffmann‐La Roche Ltd., and Novartis AG, has received payment for expert testimony (Bayer AG), and has received clinical trial support (study medicines) from Bayer AG and Laboratoires Théa.

## Supporting information


**Figure S1.** Pre‐specified criteria for defining non‐response, recurrence and treatment interval shortening, maintenance and extension in the treat‐and‐extend protocol. BCVA = best‐corrected visual acuity; CRT = central retinal thickness; IRF = intraretinal fluid; OCT = optical coherence tomography; PED = pigment epithelial detachment; SRF = subretinal fluid.

**Figure S2.** Clinical outcomes in aflibercept 8 mg treated eyes with wet age‐related macular degeneration non‐responding to aflibercept 2 mg or faricimab. Data is given as mean ± 95% confidence interval. Final timepoint = 17 eyes had 9‐month, and 21 eyes had 1‐year follow‐up. BCVA = best‐corrected visual acuity; CRT = central retinal thickness; IOP = intraocular pressure. * = *p* < 0.05; ** = *p* < 0.001.

**Figure S3.** Optical coherence tomography scans demonstrating fluid resolution following the switch to aflibercept 8 mg.

**Figure S4.** Clinical outcomes in aflibercept 8 mg treated eyes with wet age‐related macular degeneration according to the anti‐VEGF agents used prior the switch. Data is given as mean ± 95% confidence interval. Final timepoint = 17 eyes had 9‐month, and 21 eyes had 1‐year follow‐up. BCVA = best‐corrected visual acuity; CRT = central retinal thickness. * = *p* < 0.05; ** = *p* < 0.001.

**Table S1.** Baseline characteristics.

**Table S2.** Baseline characteristics.
