# Supplementary material for: Early high‐dose aflibercept 8 mg clinical outcomes in wet age‐related macular degeneration patients resistant to aflibercept 2 mg or faricimab
Source: Acta Ophthalmol. 2026 Aug 3;104(7):e885–7. doi: 10.1111/aos.70213 (PMC13651925; doi:10.1111/aos.70213)
Supplement: Supplementary file 1 [file AOS-104-e885-s001.docx]

Supplement Table 2. Baseline characteristics.

| Parameter | Aflibercept 2 mg  (N=18) | Faricimab  (N=20) | P-value |
| --- | --- | --- | --- |
| Age (years) | 79.1 ± 5.9 | 83.0 ± 5.5 | **0.047** |
| Male:Female (%) | 56:44 | 35:65 | 0.203 |
| BCVA (ETDRS letters) | 64.9 ± 13.2 | 63.3 ± 15.0 | 0.784 |
| IOP (mmHg) | 11.1 ± 2.4 | 11.9 ± 4.1 | 0.461 |
| Macular status |  |  |  |
| CRT (µm) | 362 ± 71 | 360 ± 109 | 0.939 |
| Intraretinal fluid (%) | 61 | 45 | 0.321 |
| Subretinal fluid (%) | 61 | 80 | 0.200 |
| Treatment interval (w) | 4.7 ± 1.1 | 5.1 ± 1.5 | 0.441 |

Data is given as mean ± SD or proportions. For two-group comparisons, qualitative data were analyzed with the two-factor χ² test, non-parametric variables with the Mann-Whitney U-test and continuous variables with the Student’s T-test. Abbreviations: BCVA – best-corrected visual acuity, CRT – central retinal thickness, ETDRS – Early Treatment Diabetic Retinopathy Study, IOP – intraocular pressure.
