# Supplementary material for: Early high‐dose aflibercept 8 mg clinical outcomes in wet age‐related macular degeneration patients resistant to aflibercept 2 mg or faricimab
Source: Acta Ophthalmol. 2026 Aug 3;104(7):e885–7. doi: 10.1111/aos.70213 (PMC13651925; doi:10.1111/aos.70213)
Supplement: Supplementary file 2 [file AOS-104-e885-s003.docx]

Supplement Table 1. Baseline characteristics.

| Parameter | (N=38) |
| --- | --- |
| Age (years) | 81.1 ± 6.0 |
| Male:Female (%) | 45:55 |
| BCVA (ETDRS letters) | 64.1 ± 14.0 |
| IOP (mmHg) | 11.5 ± 3.3 |
| Macular status |  |
| CRT (µm) | 361 ± 92 |
| Pigment epithelial detachment (%) | 92 |
| Drusenoid | 1 out of 35 |
| Fibrovascular | 17 out of 35 |
| Serous | 17 out of 35 |
| Intraretinal fluid (%) | 53 |
| Subretinal fluid (%) | 71 |
| Treatment interval (w) | 4.9 ± 1.3 |

Data is given as mean ± SD or proportions. Abbreviations: BCVA – best-corrected visual acuity, CRT – central retinal thickness, ETDRS – Early Treatment Diabetic Retinopathy Study, IOP – intraocular pressure.
